# Combining biosensor and metabolic network optimization strategies for enhanced l-threonine production in *Escherichia coli*

**DOI:** 10.1186/s13068-025-02640-7

**Published:** 2025-03-26

**Authors:** Zhenqiang Zhao, Rongshuai Zhu, Xuanping Shi, Fengyu Yang, Meijuan Xu, Minglong Shao, Rongzhen Zhang, Youxi Zhao, Jiajia You, Zhiming Rao

**Affiliations:** 1 Key Laboratory of Industrial Biotechnology, Ministry of Education, School of Biotechnology, Jiangnan University, Wuxi, 214122 Jiangsu China; 2 Institute of Future Food Technology, JITRI, Yixing, 214200 China; 3 College of Biochemical Engineering, Beijing Union University, Beijing, 100023 China

**Keywords:** l-Threonine biosensor, Directed evolution, High-throughput, Multi-omics analysis, In silico simulation

## Abstract

l-threonine is an integral nutrient for mammals, often used in animal feeds to enhance growth and reduce breeding costs. Developing l-threonine engineered strains that meet industrial production specifications has significant economic value. Here, we developed a biosensor that monitors l-threonine concentration to assist in high-throughput screening to capture high-yielding l-threonine mutants. Among them, the P_*cysK*_ promoter and CysB protein were used to construct a primary l-threonine biosensor, and then the CysB^T102A^ mutant was obtained through directed evolution resulting in a 5.6-fold increase in the fluorescence responsiveness of biosensor over the 0–4 g/L l-threonine concentration range. In addition, the metabolic network of mutant was further optimized through multi-omics analysis and in silico simulation. Ultimately, the THRM13 strain produced 163.2 g/L l-threonine, with a yield of 0.603 g/g glucose in a 5 L bioreactor. The biosensor constructed here could be employed for iterative upgrading of subsequent strains, and these engineering strategies described provide guidance for other chemical overproducers.

**Graphical Abstract:**

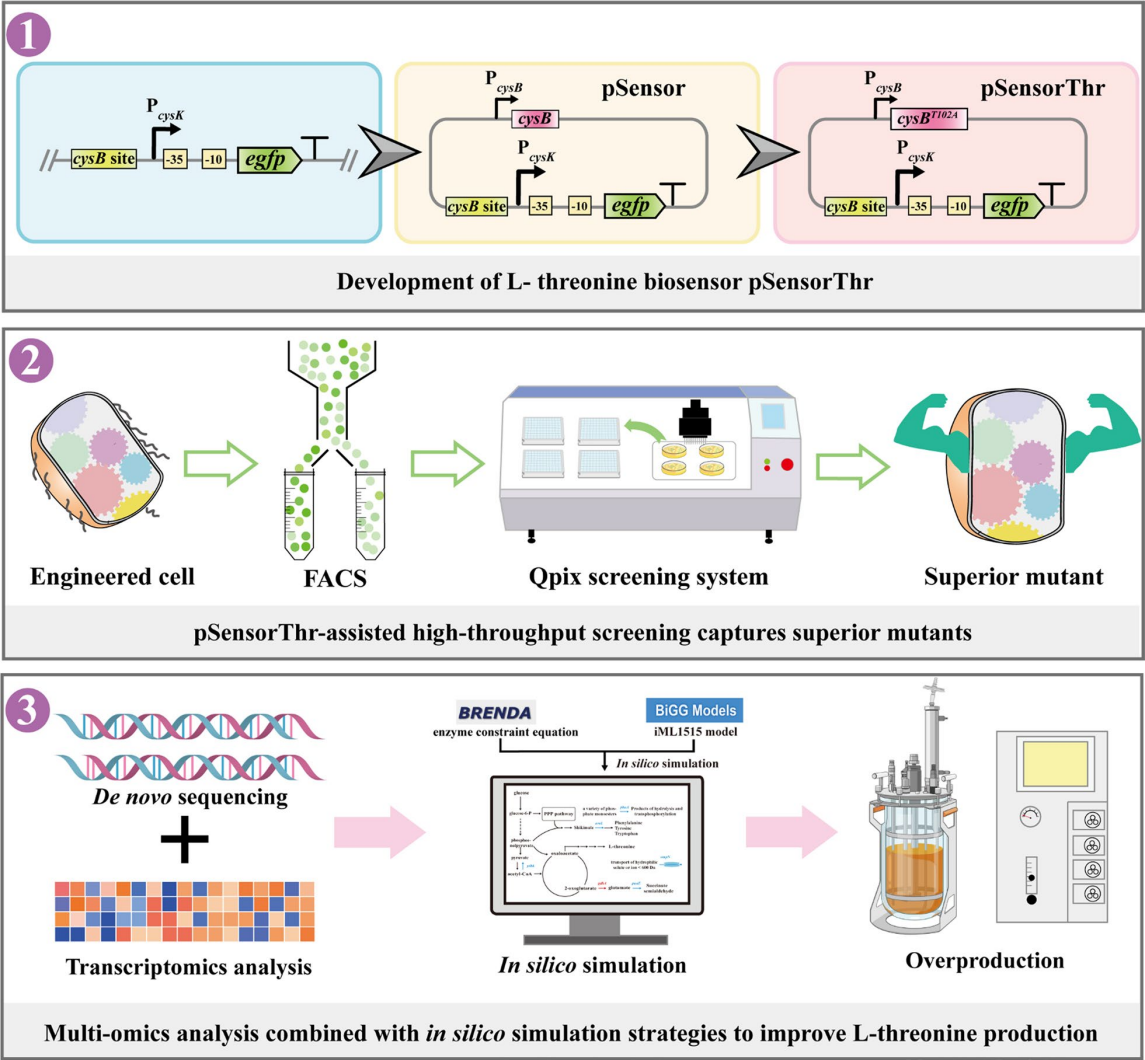

**Supplementary Information:**

The online version contains supplementary material available at 10.1186/s13068-025-02640-7.

## Background

As an essential nutrient, l-threonine is mainly used in additives, food products, and pharmaceuticals [1]. In recent years, the crucial role of l-threonine in animal feeds (such as piglet feeds) has become evident, making the development of l-threonine overproducing strains economically significant. Despite the titer of l-threonine exceeding 120 g/L in *E. coli* (Table S1) [2], the current production levels fall short when compared to l-lysine and l-glutamate [3]. With the gradual advancement in the understanding of metabolic networks, constructing microbial cell factories using systems metabolic engineering strategies to produce target chemicals has shown great promise [4, 5]. In particular, in the breeding of amino acid-producing industrial microorganisms, many reprogramming strategies have been employed to swiftly de novo develop amino acid overproducers. Although some strains have achieved significant success, their application to industrial production still faces many challenges. For example, products with high titers but low yields fail to meet the cost-effectiveness required for industrial production.

A constrained understanding of complex metabolic networks hinders the rapid identification of effective engineering targets for improving the yield of target products. Combining high-throughput screening techniques with non-rational engineering strategies has become an effective approach to achieving iterative strain evolution and efficient screening [6–8]. The utility of transcription factor biosensors, responsive to changes in specific intracellular metabolite concentrations, has become an effective practice in screening amino acid overproducers from mutant libraries [9–11]. Within the microorganism genome, the non-coding region sequence orchestrating intricate metabolic regulation serves as the natural library for these transcription factor biosensors [12, 13]. This innovative approach harnesses the intrinsic regulatory elements within the genome, offering a diverse range of potential biosensors. In addition, integrating transcriptomic analysis techniques into the screening of target biosensors, combined with rational design strategies, has paved the way for the development of more sensitive and effective identification systems [14, 15]. For example, Wu et al. pioneered the development of a biosensor device designed to respond to glucose-6-phosphate, efficiently modulating the synthesis of *N*-acetylglucosamine [16]. Liu introduced an in vivo mutagenesis system that disrupts DNA repair and replication, facilitating efficient and broad-spectrum iterative mutagenesis (MP6s) at the laboratory scale [17, 18]. These advancements in biosensor technology and screening methodologies enhance the precision and efficiency of strain construction.

In addition to the engineering strategies outlined earlier, an alternative approach to bolster strain productivity involves the exploration of effective genetic targets within the intricate global metabolic networks. For example, quantifying metabolic differences among different hosts through multi-omics analysis techniques to identify potential metabolic targets [19]. The optimal solution of metabolic fluxes is simulated in silico for maximizing the target product production. However, this approach requires optimizing the genome-scale metabolic network (GSMN) by removing specific catalytic reactions or incorporating new constraint equations to improve the accuracy of the simulation results [20].

In this study, we present engineering strategies to further increase the productivity of l-threonine producer. First, transcriptomic analysis was used to screen promoters that can sense exogenous l-threonine in *E. coli*. Next, a highly sensitive fluorescent reporter system responsive to changes in l-threonine concentration was constructed by combining CysB mutants with eGFP. Subsequently, the biosensor was used to assist the two-step high-throughput screening technique for strain iterative evolution to capture superior mutants. Next, beneficial targets are identified through multi-omics analysis, and intracellular carbon flux allocation is further optimized using GSMN to maximize l-threonine production. This study not only developed an l-threonine overproducing strain with potential industrial applications but also demonstrated a novel approach for constructing highly sensitive l-threonine biosensors, offering new avenues for the development of biosensors targeting other chemicals.

## Materials and methods

### Strain, plasmids, chemicals

Transcriptomic and genomic analysis, Oligonucleotides synthesis were implemented by Azenta (Suzhou, China). Multif seamless assembly mix were supplied by AB clonal (Wuhan, China). DNA polymerases (p525); gel DNA, plasmid, and genome extraction kits were produced by Vazyme (Nanjing, China). The strains and plasmids used in this study can be found in Supplementary Table S2.

### Transcriptomics analysis of gene expression in response to l-threonine addition

An individual colony of wild-type MG1655 was inoculated in 10 mL high-salt Luria–Bertani (LB) medium (10 g/L tryptone, 5 g/L yeast extract, 10 g/L NaCl) for 12 h (220 rpm, 37 °C). 2 mL of culture was injected into 100 mL LB medium for 4 h and then all was transferred to 500 mL flasks containing 0, 30, and 60 g/L l-threonine, respectively. After continuing the incubation for 2 h, all cultures were harvested and centrifuged at 8000×*g*, 4 °C, for 10 min. After removing the supernatant, the tubes containing the cells were immediately placed in dry ice to ensure rapid freezing and prevent RNA degradation. Subsequently, these samples were shipped to Azenta (Suzhou, China) for transcriptomic analysis.

### Transcriptomics analysis of THRM1 and THR36-L19 strains

Individual colonies of THRM1 and THR36-L19 were inoculated in 10 mL LB medium for 12 h (220 rpm, 37 °C), respectively. 2 mL cultures of THRM1 and THR36-L19 were injected into 100 mL LB medium for 10 h (220 rpm, 37 °C). Then, all cultures of THRM1 and THR36-L19 were transferred to a 5 L bioreactor containing 2 L fermentation medium (composition detailed in the “Fermentation conditions in shake flasks” section), respectively. The dissolved oxygen (DO) was maintained at 30%, and the pH was maintained at 7.0 by increasing the stirring speed and adding ammonia. After 14 h, 50 mL cultures of THRM1 and THR36-L19 were harvested, and each was centrifuged at 8000×*g*, 4 °C for 10 min. After removing the supernatant, the cultures of THRM1 and THR36-L19 were submitted to Azenta (Suzhou, China) for transcriptomics analysis (stored in dry ice).

### De novo sequencing

An individual colony of the THRM1 strain was inoculated in 10 mL LB medium for 12 h (220 rpm, 37 °C). Then, 2 mL culture was injected into 100 mL LB medium for 12 h. All cultures were harvested and centrifuged at 8000×*g*, 4 °C, for 10 min. After removing the supernatant, the culture was submitted to Azenta (Suzhou, China) for genome sequencing (stored in dry ice). DNA sequence analysis was conducted using the genome of the model strain *E. coli* MG1655 as a reference.

### Design and refinement of l-threonine biosensors

The complete non-coding regions of 21 genes were amplified by PCR using the *E. coli* genome as a template. Then, the non-coding region sequences, linearised pTrc99A and eGFP were ligated using the Seamless Cloning Kit and the reaction products were all transferred to *E. coli* DH5α and cultured on LB agar plates for 12 h at 37 °C to construct the initial fluorescent reporter system. To validate the system, *E. coli* DH5α cells were transformed with an eGFP plasmid driven by no promoter as a negative control to establish a fluorescence baseline. In addition, *E. coli* DH5α cells were transformed with an eGFP plasmid under the control of the strong constitutive promoter J23119 as a positive control to ensure the proper functioning of the fluorescence reporter system (Fig. S1). Positive transformants were selected from the plates and inoculated into 24-well plates containing LB medium with 0, 10, 20, and 30 g/L l-threonine. The cultures were incubated for 8 h (220 rpm, 37 °C). Then, the eGFP fluorescence of the transformants in different concentrations of l-threonine was measured, and plasmids with a linear positive response were screened. P_*cysK*_, P_*cysJ*_, P_*cysP*_, and P_*cysD*_ truncation mutants were obtained by reverse amplification of the parental plasmid using primers deleting the CysB binding site. The plasmid linking the P_*cysK*_*-egfp* gene was linearized and then tandemly linked to the CysB protein to construct the pSensor biosensor. Subsequently, the pSensor plasmid was reverse amplified using primers containing the point mutation to get the pSensorThr mutant containing CysB^T102A^.

### RBS library preparation and screening

The first 210 bp of the *gdhA* gene was selected to be ligated to the *egfp* gene using a flexible linker (GGGGS)_3_ and integrated onto the pTrc99A plasmid to construct a fluorescent reporter system. The parental plasmid was used as a template and amplified by primers containing 10 degenerate bases “N” (any one of A, T, G, C) for the construction of the RBS mutant library (Table S3). The PCR products were digested using *Dpn* I enzyme for transformation into *E. coli* DH5α and the cells were cultured on LB agar plates at 37 °C for 12 h. Single clones were selected from the plates and inoculated into 24-well plates, then incubated for 10 h (200 rpm, 37 °C). In a 24-well plate, the P_*gdhA*_ promoter-driven *gdhA* (210 bp)-*egfp* plasmid was used as a negative control, while the expression of *gdhA* (210 bp)-*egfp* driven by the P_*trc*_ promoter and the original RBS was used as a positive control. Strains with different fluorescence expression intensities were selected from 110 mutants for sequencing analysis. Following incubation, they were centrifuged at 8000×*g* for 10 min, and the pellets were suspended and washed twice with phosphate-buffered saline (PBS). Then, the mutants with higher fluorescence than the parental plasmid were screened from the library. The fluorescence output of cells was detected using a multifunctional enzyme marker Biotek (synergy H4).

### Screening l-threonine overproducers from mutation libraries

First, the MP6^ts^ plasmid was transformed into competent cells of the THR36-L19 strain to generate THR36-L19-MP6^ts^. A single clone of positive transformant was inoculated in LB medium and induced with 0.1 mM arabinose for 24 h (220 rpm, 30 °C) to generate mutant library. Removal of the MP6^ts^ plasmid from the mutant was achieved by incubation at 42 °C. Transforming the pSensorThr plasmid into competent cells of mutant library was for screening l-threonine overproducing strains. Mutants containing the pSensorThr plasmid were inoculated into 50 mL fermentation medium for 12 h to accumulate l-threonine and induce eGFP expression. 2 mL culture was washed three times with PBS and resuspended to an OD_600_ of 0.4 for flow cytometry analysis. Based on forward scatter and side scatter signals, gating was performed to minimize interference from erroneous events. After gating, at least 20,000 events were collected for subsequent analysis, and the top 0.5% of cells with the highest fluorescence intensity were sorted for further sorting and fluorescence measurement.The enriched highly fluorescent population was coated on LB plates and incubated for 20 h at 37 °C. Then, 190 clones were inoculated into two 96-deep well plates with fermentation medium by Qpix 420 (Molecular Devices) for 12 h and then fluorescence was determined. From these, the top 10 mutants with the highest fluorescence values were selected and the above operation was repeated for iterative mutation of the strains. After five rounds of random mutagenesis, the 50 strains with the highest fluorescence values were selected for validation of fermentation performance in shake flasks. The production of l-threonine was determined by high-performance liquid chromatography.

### Carbon flux balance analysis and target prediction for gene knockout

A model based on *E. coli* iML1515 (http://bigg.ucsd.edu/models/iML1515) was further modified for Flux Balance Analysis (FBA) and Optknock analysis experiments. The model incorporated the PYC_Re_-catalyzed oxaloacetate synthesis pathway, introducing the compound reaction equation: ATP + pyruvate + HCO_3_^−^ + H^+^  → ADP + phosphate + oxaloacetate. In addition, the reaction fluxes of *tdcC*, *tdh*, *sstT*, *ilvA*, *poxB*, *pflB*, *pykF*, and *ldhA* genes were tuned to zero in the model. Further enhancement of l-threonine synthesis was achieved through the modification GSMN model with OptKnock, a computational framework for predicting knockout targets to achieve chemical overproduction [21]. The l-threonine synthesis reaction was designated as the target function. The in silico to simulation solution was implemented using COBRA Toolbox (v3.0) [22] and Gurobi Optimizer. The CRISPR/Cas9 gene editing system was used to knockout genes predicted by in silico analysis, following the experimental procedure outlined by Zhao et al. [23]. Shake flask fermentation was then employed to assess the impact of the gene knockouts on strain growth and l-threonine production.

### Fermentation conditions in shake flasks

For shake flask incubation, a single colony was selected from LB agar medium and inoculated in a 50 mL shake flask containing 10 mL LB medium at 220 rpm, 37 °C. After 12 h feeding, 2 mL culture was transferred to a 500 mL shake flask with 28 mL of seed medium. The seed medium composition included 15 g/L corn steep liquor, 3 g/L yeast extract, 2 g/L citrate, 0.5 g/L MgSO_4_·7H_2_O, 20 mg/L FeSO_4_·7H_2_O, 20 mg/L MnSO_4_·H_2_O, and 30 g/L glucose. Following an additional 10 h of feeding, 5 mL culture was shifted to a 500 mL shake flask with 25 mL fermentation medium. The fermentation medium contained 10.0 g/L corn steep liquor, 2.0 g/L yeast extract, 2.0 g/L citrate, 1.0 g/L KH_2_PO_4_, 1.0 g/L MgSO_4_·7H_2_O, 20.0 mg/L FeSO_4_·7H_2_O, 20.0 mg/L MnSO_4_·H_2_O, 3 g/L (NH_4_)_2_SO_4_, 0.2 mg/L biotin, 0.2 mg/L vitamin B1, 0.02 mg/L vitamin B12, and 15 g/L glucose. The seed medium is used during the initial cell expansion phase, while the fermentation medium is used after cell expansion for the fermentation phase to achieve efficient metabolic product synthesis.

### Fed-batch fermentation in a 5 L bioreactor

A single colony of positive bacteria was harvested from LB agar medium and inoculated into 10 mL LB medium. After a 12-h incubation period, 2 mL culture was transferred to a 500 mL shake flask containing 100 mL LB medium and cultivated for 10 h. Then, 100 mL culture was transferred to a 5 L bioreactor with 1.9 L seed medium and cultivated for an additional 10 h at 37 °C. The cultivation continued until glucose was completely consumed. DO levels were controlled at 30.0% ± 2.0% by automatically adjusting the stirring speed, while the pH was maintained at pH 7.0 ± 0.02 through the automatic addition of ammonia. Following this, 400 mL the culture (20% inoculum) was transferred to a 5 L bioreactor filled with a 1.6 L fermentation medium. The parameters for DO and pH during fermentation were kept consistent with the previous stage.

### Analytical methods

The biomass of the strain was detected by measuring the optical density at 600 nm using the UV-1200 spectrophotometer from AOE INSTRUMENTS, Shanghai, China. Glucose concentration in the fermentation broth was quantified by the Biosensor Analyzer S-10 from Sieman Technology, Shenzhen, China. Amino acids and organic acids in the fermentation broth were detected via high performance liquid chromatography (HPLC) utilizing pre-column derivatization with *o*-phthaldialdehyde and elution with 5 mM H_2_SO_4_, respectively [19]. l-threonine assay: Mobile phase A: 8 g sodium acetate dissolved in 1000 mL water, 225 μL triethylamine added, pH adjusted to 7.20 ± 0.05 with acetic acid, then 10 mL tetrahydrofuran added. Mobile phase B: 6 g sodium acetate dissolved in 200 mL water, pH adjusted to 7.20 ± 0.05 with acetic acid, then 400 mL methanol and 400 mL acetonitrile added. Column type: C_18_ column (250 × 4.6 mm); Column temperature: 40℃; Flow rate: 1 mL/min; UV detection wavelength: 338 nm; Gradient elution: 0–17.5 min: from 92% A, 8% B to 40% A, 60% B; 17.5–21.5 min: from 40% A, 60% B to 100% B; 21.5–24 min: 100% B; 24–30 min: from 100% B to 92% A, 8% B; Run time: 30 min. The FCS3 format data obtained were processed using FlowJo_V10 software. Experimental data were expressed as mean ± standard deviation on at least three parallel experiments.

## Results and discussion

### Screening for endogenous genetic elements responsive to changing l-threonine levels

With the continuous advancement of synthetic biology technology, the establishment of microbial cell factories for the production of target chemicals has become a popular direction [24, 25]. Alongside rational modification strategies, screening for phenotypes of interest from large mutation libraries is another viable approach for acquiring high-yielding strains [10, 26]. Multi-omics technology is an effective tool for rapidly mining relevant genetic factors from microorganisms to construct biosensors for target products [27, 28]. Jiang et al. successfully constructed an arginine overproducing strain using a biosensor-assisted high-throughput screening platform [29]. Liu et al. constructed a fusion promoter, P_*cysJH*_, for screening l-threonine mutants by proteomic analysis [30]. Inspired by this, we endeavored to develop a highly sensitive l-threonine biosensor for efficient screening.

**Fig. 1:**
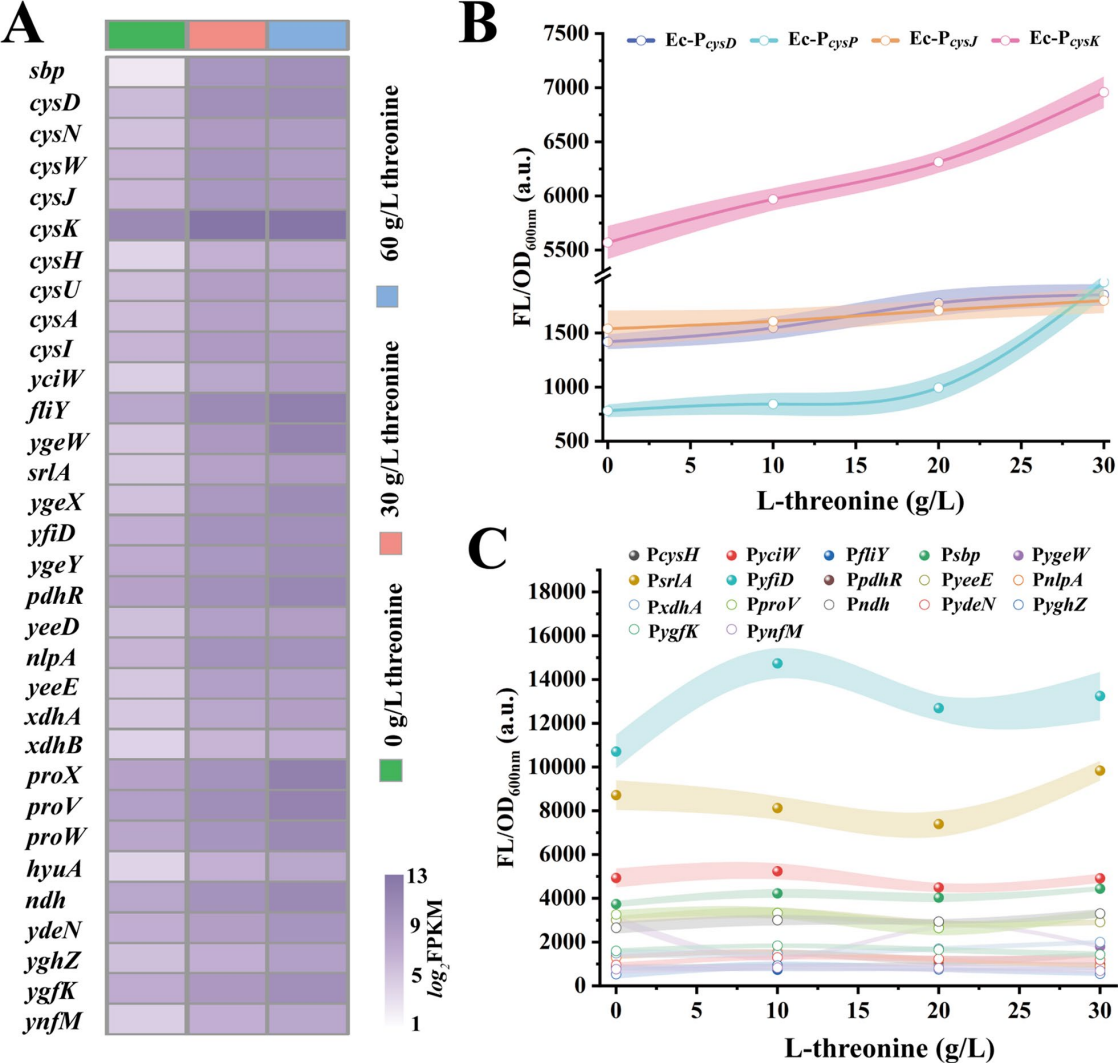
Mining, design and characterization of l-threonine biosensor. **A** Cluster analysis of gene expression. Cluster analysis with linear positive correlation of gene expression levels in *E. coli* MG1655 after treatment with 0, 30, and 60 g/L l-threonine.** B** Fluorescent reporter system was constructed to characterize the changes in green fluorescence intensity of cells after induction of P_*cysK*_, P_*cysJ*_,P_*cysD*_,P_*cysP*_ promoters at different concentrations of l-threonine (0, 10, 20 and 30 g/L). **C** To characterize the changes of green fluorescence intensity of cells after inducing the remaining 17 promoters with different concentrations of l-threonine (0, 10, 20 and 30 g/L)

To mine genetic coding elements in *E. coli* that could respond to changes in l-threonine concentration to construct a primary fluorescent reporter system, *E. coli* MG1655 was induced by exogenous addition of l-threonine at different concentrations (0, 30, and 60 g/L). Subsequently, the global gene expression changes were investigated by transcriptomics analysis, and 32 genes that were positively correlated with the l-threonine concentration were preliminarily screened based on their expression (Fig. 1A). The positional distribution of the 32 genes in the genome was investigated and it was found that some of the genes were in the same operon (such as *cysDN*, *cysPUWA*, *cysJI*, *ygeWXY* and *hyuA*, *yeeED*, *xdhAB*, *proVWX*), so the promoters of 21 of these genes were further screened and characterized. The non-coding regions of these 21 genes were ligated in tandem with the eGFP protein on the pTrc99A plasmid to create promoter library. Then, the responsiveness of these promoters at 0, 10, 20 and 30 g/L l-threonine levels was examined. Among them, P_*cysD*_, P_*cysJ*_, P_*cysP*_, and P_*cysK*_ showed a linear positive correlation with l-threonine concentration and the P_*cysK*_ promoter showed the best response performance (Fig. 1B), whereas the other promoters did not show a significant correlation (Fig. 1C). Although the ability of the ‘*cys*’ promoter to respond to l-threonine was determined by further characterization of the promoter library, the narrow range of fluorescence thresholds produced by this natural genetic element tends to lead to a high rate of false positives during the screening process.

### Engineering natural sensors to improve response to l-threonine

**Fig. 2:**
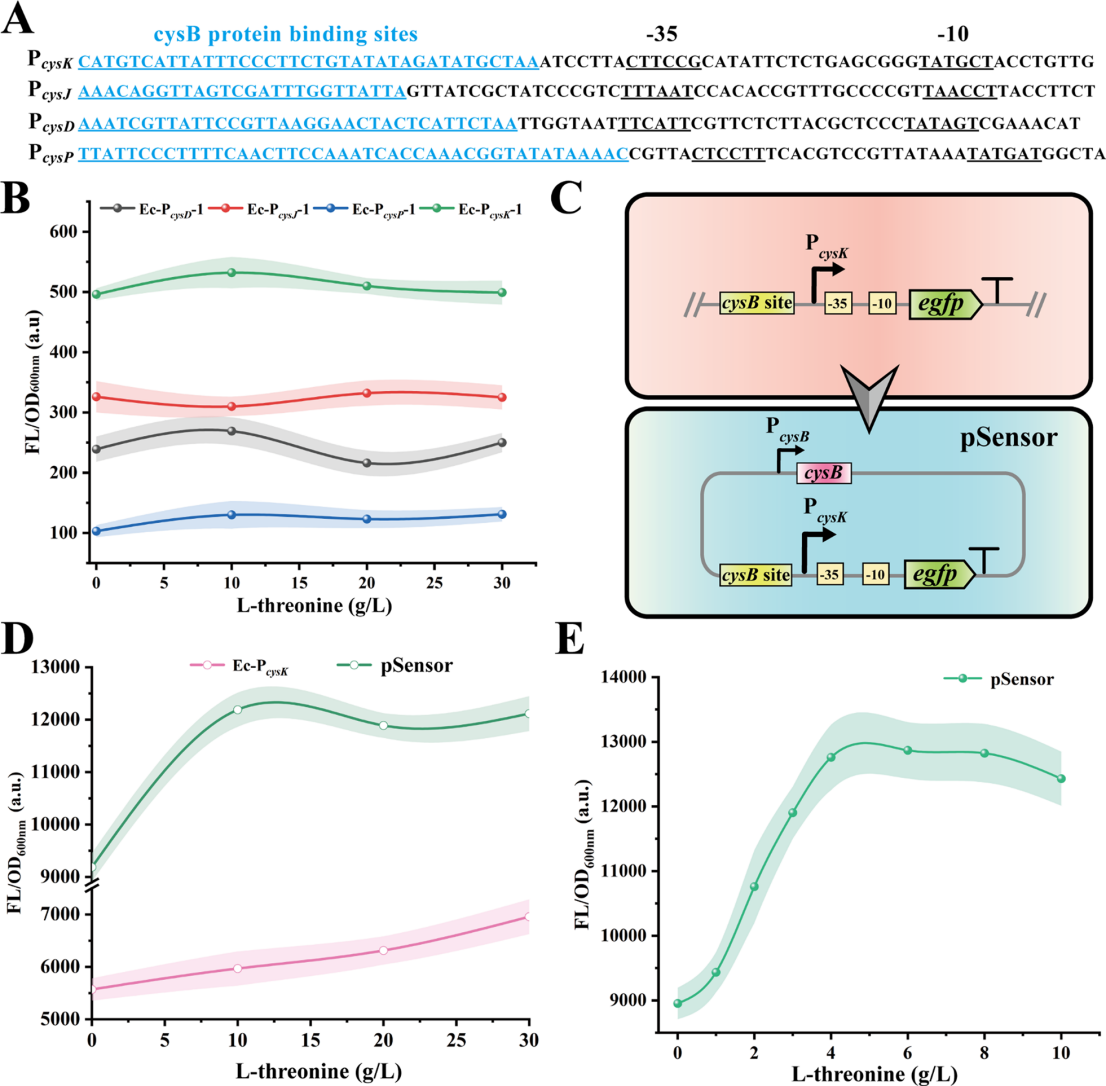
Development, test and optimization of the l-threonine biosensor. **A** Sequence analysis of promoters. Sequence analysis of P_*cysK*_, P_*cysJ*_, P_*cysD*_, and P_*cysP*_ promoters, highlighting the binding site of CysB protein.** B** Fluorescence values of the sensor at 0, 10, 20, and 30 g/L l-threonine concentration after deletion of the binding site of CysB protein in promoters P_*cysD*_, P_*cysJ*_, P_*cysP*_, and P_*cysK*_.** C** Schematic diagram of the pSensor biosensor structure.** D** Characterization of the performance of biosensors pSensor and P_*cysK*_-egfp. The *x*-axis represents different concentrations of l-threonine, and the y-axis represents the change in intracellular green fluorescence intensity after induction. **E** Identification of the response range of the biosensor pSensor to l-threonine

To address the issues of low sensitivity and response threshold in natural sensors, an artificial design was implemented to enhance screening performance. Initially, non-coding region sequences from *cysD*, *cysJ*, *cysP*, and *cysK* genes were compared (Fig. 2A), revealing the presence of CysB protein binding sites upstream of the -35 region [31, 32]. We speculated that the responsiveness of the “*cys*” promoters to l-threonine might be influenced by the CysB protein. Therefore, the CysB binding site in the P_*cysD*_, P_*cysJ*_, P_*cysP*_, and P_*cysK*_ promoters were deleted to produce Ec-P_*cysD*_-1, Ec-P_*cysJ*_-1, Ec-P_*cysP*_-1, and Ec-P_*cysK*_-1, respectively. Testing revealed that these mutants lost their responsiveness to l-threonine and displayed only background expression (Fig. 2B). Subsequently, the biosensor “pSensor” was constructed by overexpressing CysB in the higher sensitive pTrc99A-P_*cysK*_-egfp plasmid (Fig. 2C). Compared to P_*cysK*_-egfp, pSensor exhibited a higher fluorescence threshold at l-threonine concentrations of 0–10 g/L but lacked correlation at 10–30 g/L (Fig. 2D). Further optimizing the induced concentration of l-threonine, the pSensor was determined to maintain a linear fluorescence response between 0 and 4 g/L l-threonine (Fig. 2E), and the fluorescence threshold was improved by about 2.1-fold compared with P_*cysK*_-egfp.

**Fig. 3:**
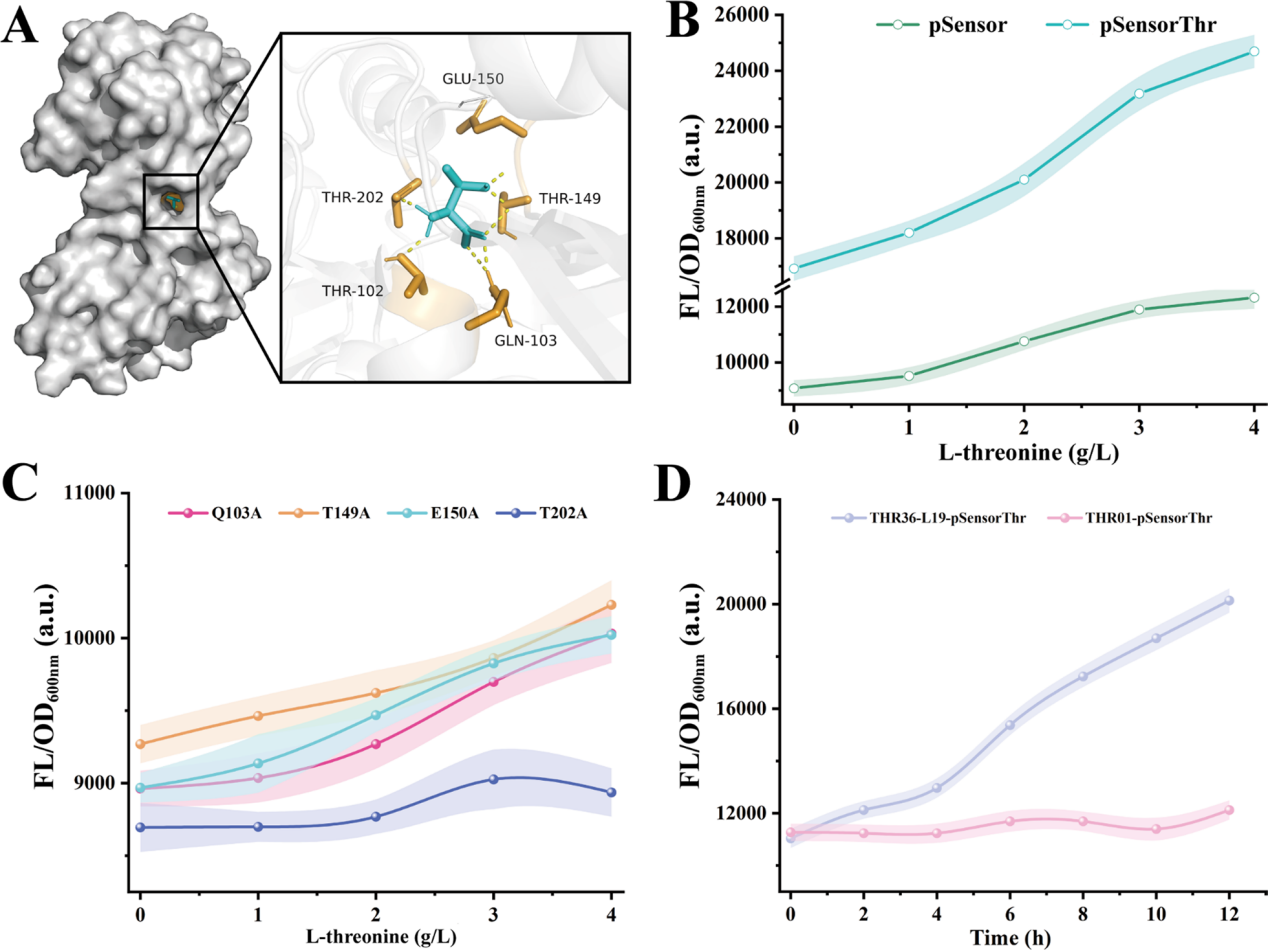
Development of pSensorThr biosensor. **A** Docking outcome between the CysB_*Ka*_ protein and l-threonine is illustrated. Hydrogen bonds are highlighted using yellow dotted lines. **B** Performance comparison of biosensors pSensor and pSensorThr. **C** Fluorescence values of the biosensor at concentrations of 0–4 g/L l-threonine after overexpression of the CysB mutant. **D** Analyzing the responsiveness of pSensorThr to endogenous l-threonine

To further investigate the effect of CysB on the biosensor, the CysB protein crystals of *Klebsiella aerogenes*, which is highly similar to CysB of *E. coli* (10.2210/pdb1al3/pdb, 93.8% amino acid sequence similarity to CysB_*Ec*_), were used for molecular docking with l-threonine. The binding of l-threonine to CysB_*Ka*_ was simulated using Autodock Vina, and the results were processed and analyzed by Pymol software. As a result, the amino acid residues T102, Q103, T149, E150, and T202 have hydrogen bonding force with l-threonine ligand (Fig. 3A). To further verify whether l- l-threonine affects P_*cysK*_ expression by interacting with CysB protein, these amino acid residues were mutated to alanine. Among them, CysB T102A led to an approximately 2.4-fold increase in the response threshold of the biosensor compared to the pSensor (named the CysB T102A mutant as pSensorThr) (Fig. 3B), whereas Q103A, T149A, E150A did not show any significant changes, and T202A led to a loss of response of P_*cysK*_ to concentration changes (Fig. 3C).

To validate the responsiveness of pSensorThr to endogenous l-threonine, we introduced the biosensor into two *E. coli* strains: one strain capable of producing a high yield of l-threonine (named THR36-L19, accumulating 2.8 g/L of l-threonine during fermentation in 24-well plates containing 10 g/L glucose), and the other being the non-producing *E. coli* THR01 strain [33]. The resulting strains were named THR36-L19-pSensorThr and THR01-pSensorThr. When incubated in 24-well plates containing fermentation medium, THR36-L19-pSensorThr exhibited a linear fluorescence response, whereas THR01-pSensorThr showed no significant change (Fig. 3D, Fig. S2). This suggested that the pSensorThr could effectively detect the endogenous l-threonine synthesis. In comparison with existing l-threonine biosensors, such as the one developed by Liu et al. [30], which fused the P_*cysJH*_ promoter to drive the expression of the eGFP reporter gene, and the Thr l-P_*trc*_ l-threonine biosensor constructed by Su et al. [34], the pSensorThr biosensor system exhibits a higher fluorescence response threshold. This broader fluorescence range helps reduce the risk of misjudgment caused by fluorescence errors during screening. However, the l-threonine concentration response threshold is lower than those of the biosensors developed by Liu (0–50 g/L) and Su (0–20 g/L). Therefore, further optimization of the response range of pSensorThr to l-threonine in future studies will enhance the applicability of this biosensor.

### Assisting high-throughput screening to identify l-threonine overproducers

**Fig. 4:**
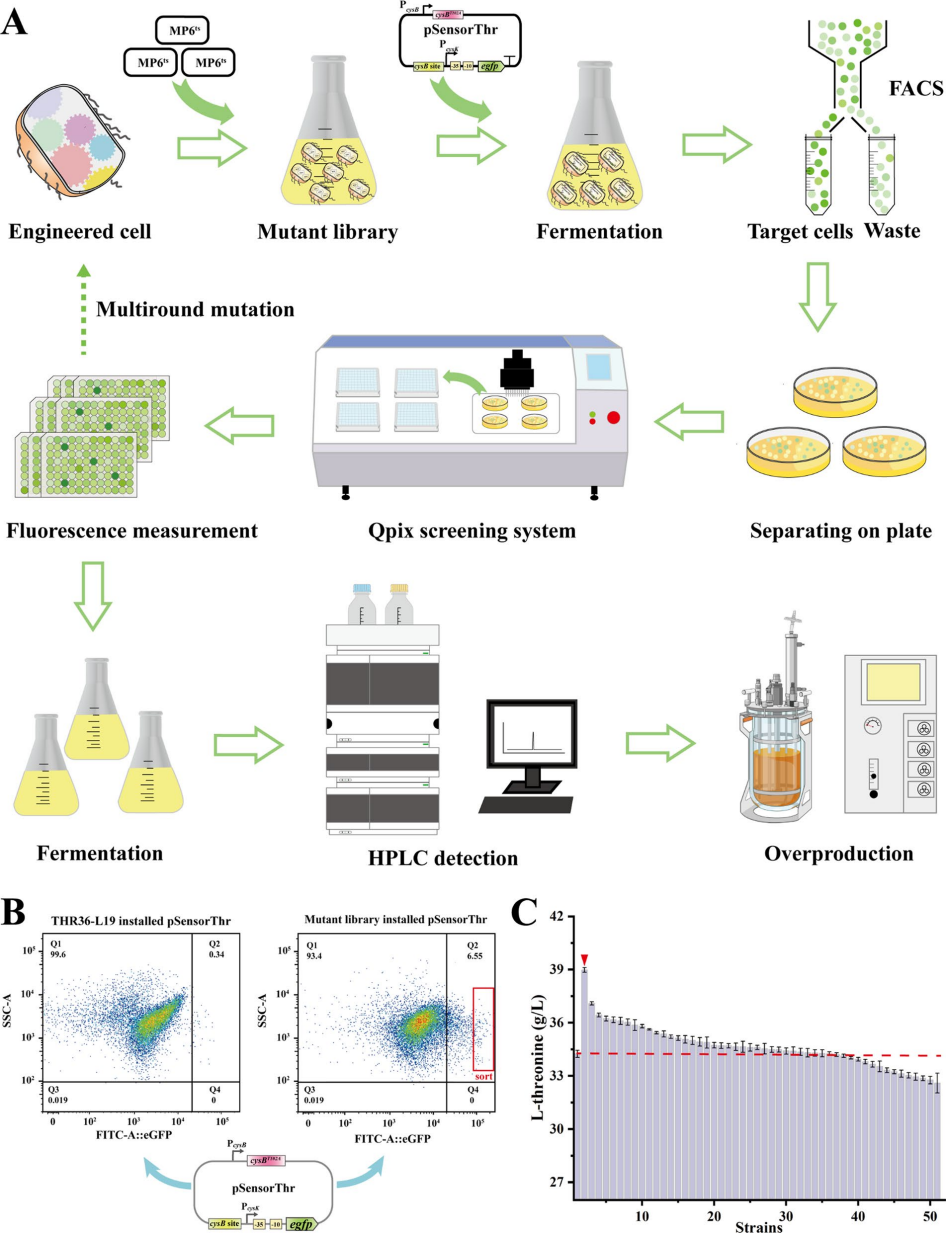
Application of pSensorThr biosensor. **A** Flowchart for the selection of l-threonine overproducers by random mutagenesis and two-step high-throughput screening. Transformation of MP6^ts^ plasmid is introduced into competent cells of the THR36-L19 strain, creating a random mutation library. The pSensorThr plasmid is then introduced into the library to screen highly fluorescent cell populations using Fluorescence-Activated Cell Sorting (FACS). The Qpix system is employed to select single target clones from the cell population. Mutants with high fluorescence levels undergo multiple rounds of mutagenesis and are tested for fermentation performance in shake flasks and 5 L bioreactors. **B** FACS screening of mutant library. Fluorescence-activated cell sorting (FACS) was performed to select cells with higher fluorescence from the mutant library with pSensorThr installation. **C** l-threonine production in mutants. l-threonine production in 50 highly fluorescent mutants was obtained by multiple rounds of random mutagenesis and high-throughput screening. THR36-L19 served as a control. l-threonine production was determined by HPLC, and data represent means and standard deviations of independent triplicates

Mutation library construction with a broad mutational spectrum is essential for screening superior mutants. To construct a large mutation library of THR36-L19 strain, the temperature-sensitive MP6^ts^ plasmid was transformed into THR36-L19 (Fig. 4A). The hosts carrying the MP6^ts^ plasmid were induced and mutated at 30 °C, and the plasmid was eliminated by incubation at 42 °C [7]. Then, shake flask fermentation was conducted after incorporating the pSensorThr biosensor into mutant libraries. Highly fluorescent cell populations were then screened using fluorescence-activated cell sorting (FACS) (Fig. 4B). Then, Qpix screening system was used for further high-throughput sorting of single cells from highly fluorescent groups. The top 10 strains with the highest fluorescence values were selected for the next round of mutation and screening. After five rounds of screening, 50 mutant strains were obtained. Subsequently, the productivity of each strain was evaluated through shake flask culture. Most demonstrated higher l-threonine accumulation compared to THR36-L19. Ultimately, the mutant *E. coli* strain THRM1 was successfully identified, accumulating 38.97 g/L l-threonine in shake flask culture (Fig. 4C).

### Multi-omics-guided reverse metabolic engineering further enhances l-threonine production

**Fig. 5:**
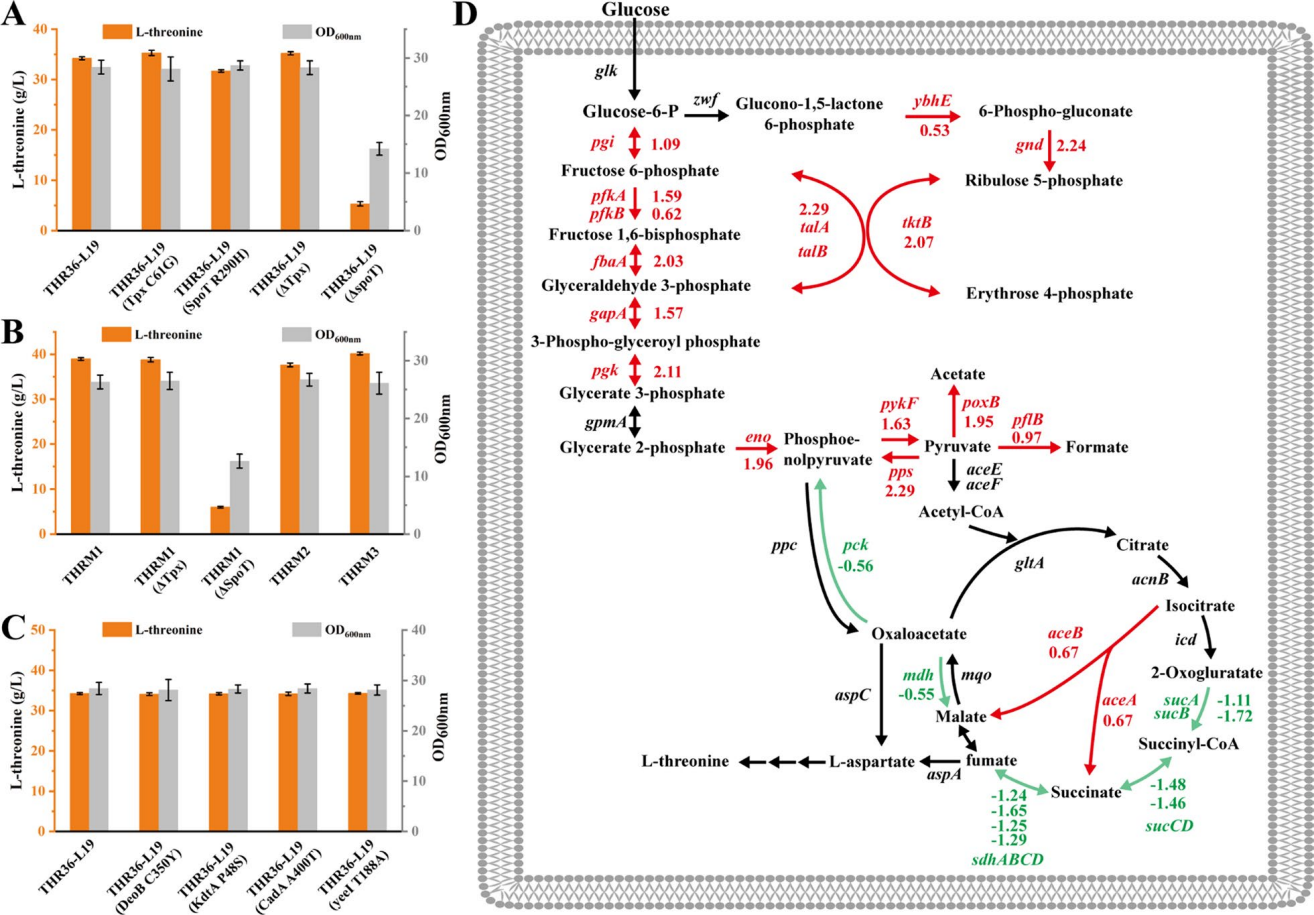
Reverse metabolic engineering guided by multi-omics analysis to explain l-threonine overproduction. **A**, **C** Identification of beneficial targets. Integration of Tpx C61G, DeoB C350Y, TrmC A129P, SpoT R290H, *cadA*^A400T^, and *yeeI*.^T188A^ mutants into THR36-L19 strain verify their effect on l-threonine production and cell growth. **B** Integration of Tpx G61C and SpoT H290R into THRM1 strain verify their effect on l-threonine production and cell growth. **D** Transcriptomic analysis. The log_2_ calculation of fragments per kilo bases per million reads for THRM1/THR36-L19 is shown. The red color indicates transcriptional up-regulation, while the green color indicates transcriptional down-regulation compared to THR36-L19

To elucidate the reason for the higher l-threonine production in THRM1 strain, multi-omics analysis (genomics and transcriptomics) was applied to clarify the genetic mechanism of the mutant [19, 29]. Genomic analysis through de novo sequencing revealed four missense mutations (Tpx C61G, DeoB C350Y, TrmC A129P, SpoT R290H) and two nonsense mutations (*cadA*^A400T^, *yeeI*^T188A^) in the coding sequences. To verify the effect of these mutants on l-threonine synthesis, these mutations were individually integrated into the original locus of the parental strain THR36-L19. The recombinant THR36-L19 (Tpx C61G) showed a 3.12% increase in l-threonine production, while THR36-L19 (SpoT R290H) exhibited a 7.41% decrease. In addition, knocking out the *tpx* gene in THR36-L19 led to a 2.91% increase in l-threonine production, whereas knocking out the *spoT* gene severely inhibited both strain growth and l-threonine production (Fig. 5A). Subsequently, the Tpx G61C and SpoT H290R mutants were individually integrated into the native loci of the THRM1 strain, resulting in THRM2 and THRM3. The l-threonine yields of these strains decreased by 3.45% (37.63 g/L) and increased by 3.1% (40.18 g/L), respectively (Fig. 5B). The l-threonine titer of strains integrating DeoB C350Y, TrmC A129P, *cadA*^A400T^, and *yeeI*^T188A^ mutants was similar to THR36-L19 (Fig. 5C). The effect of the combined mutants on l-threonine production could be further investigated in subsequent experiments.

Transcriptomic analysis of THRM1 and THR36-L19 strains revealed upregulation of genes involved in the EMP pathway and glyoxylate bypass were upregulated, while most genes in the TCA cycle were downregulated (Fig. 5D). This suggested faster synthesis of l-threonine from glucose in the THRM1 strain. Among these pathways, the expression level of the *pps* gene encoding phosphoenolpyruvate synthase (PPS) increased by 4.89-fold, facilitating PEP replenishment. Upregulation of *pykF*, *poxB,* and *pflB* genes were observed, potentially reducing carbon fluxes into the targeted metabolic pathways. By sequentially knocking out the *pykF*, *poxB*, and *pflB* genes in the THRM3 strain, the THRM4, THRM5, and THRM6 strains were generated, respectively. Among them, THRM6 achieved an l-threonine yield of 41.36 g/L (Fig. S2).

Genomics analysis and reverse metabolic engineering revealed that the Tpx C61G mutant and SpoT H290R revertant played pivotal roles in enhancing l-threonine production in THRM1. RNA-seq further unveiled significant metabolic differences between THRM1 strain and its parental strains. The up-regulation of the EMP pathway and the glyoxylate cycle, coupled with the down-regulation of the TCA cycle, indicated that THRM1 efficiently utilized glucose and produced l-threonine through a more optimal pathway. In addition, transcriptomic analysis revealed that up-regulation of the expression of *pykF*, *poxB*, and *pflB* genes resulted in leakage of some carbon sources. Although we identified new engineered targets to promote l-threonine synthesis, the regulatory mechanism leading to the increased yield was not resolved. An in-depth study of the genetic differences between strain THRM1 and THR36-L19 could be instrumental in further enhancing l-threonine production.

### Maximizing l-threonine production by optimizing metabolic flux through in silico simulation

The complexity of metabolic target screening presents challenges in metabolic engineering [35]. In silico simulation of intracellular metabolic flux allocation and optimization using GSMN is an emerging approach in metabolic engineering [36]. Among these simulation methods, optKnock is a computational technique designed to predict knockout genes that balance cell growth and maximize product production [21].

**Fig. 6:**
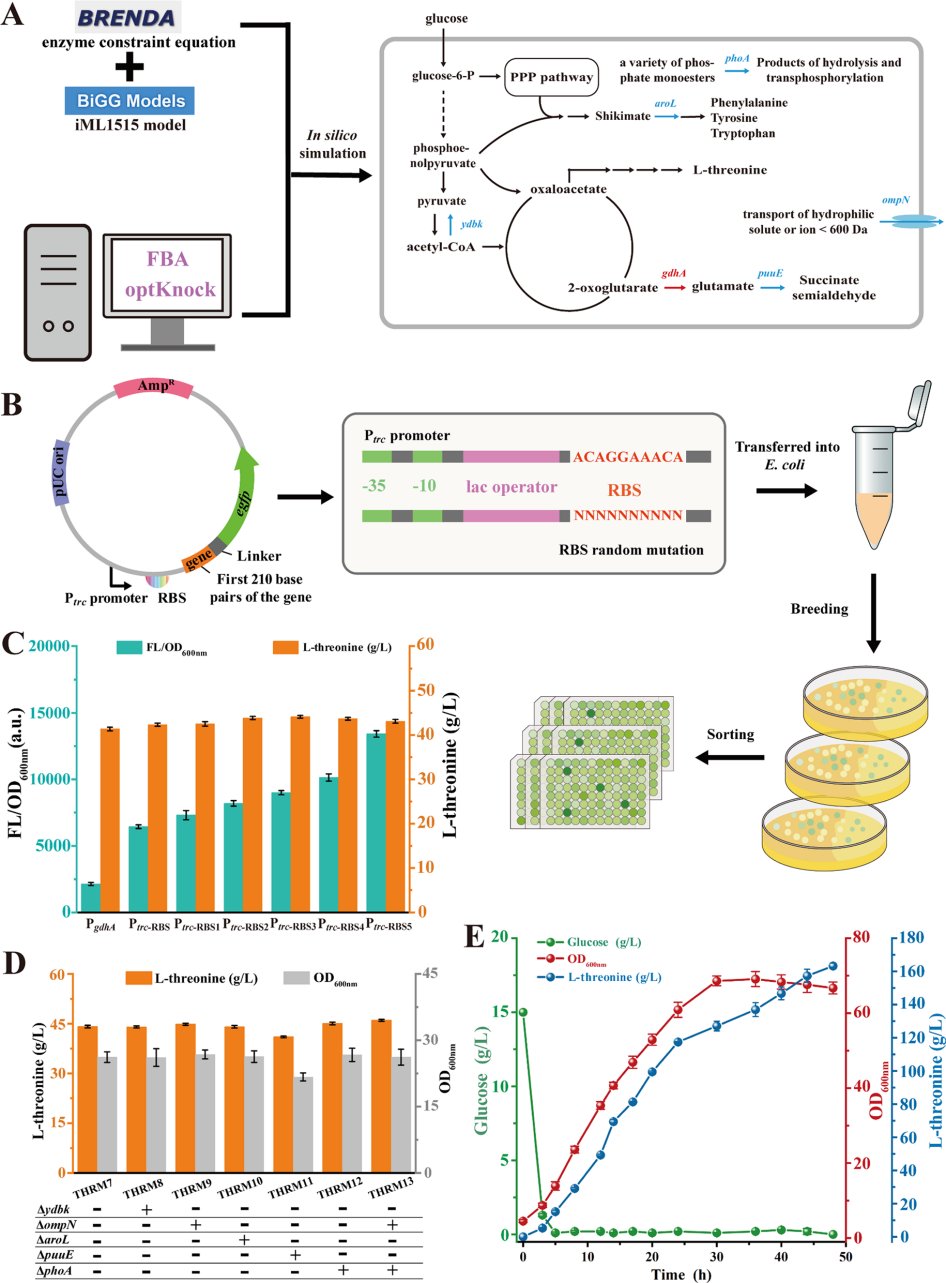
Predicting new metabolic engineering targets in silico. **A** Flux balance analysis and prediction of knockout targets. Flux balance analysis and prediction of new knockout targets using an expanded GSMN. **B** RBS library construction and screening. The first 210 bp of the target gene (*gdhA*) is fused with the *egfp* gene using a flexible linker (GGGGS)_3_ and inserted behind the P_*trc*-RBS_ promoter. RBS random mutation libraries are constructed using primers containing degenerate bases. Transformants are cultured in 96-well plates for further screening based on green fluorescence.** C** Fluorescence analysis of P_*trc*-RBS_ mutants. Analysis of fluorescence in P_*trc-*RBS_ mutants using the *gdhA-egfp* fusion gene as a reporter gene. The effect of replacing the native promoter of *gdhA* gene with P_*trc*_-_RBS_ mutants on l-threonine production is assessed. **D** Effect of optKnock-predicted target gene knockout. The effect of optKnock-predicted target gene knockout on l-threonine synthesis and cell growth is evaluated. **E** Fermentation parameters in a 5 L bioreactor. Fermentation parameters of the THRM13 strain in a 5 L bioreactor are presented for further analysis

To identify new metabolic targets for enhancing l-threonine production, the GSMN model iML1515 of *E. coli* guided the metabolic engineering of the THRM6 strain [37]. Initially, the reaction equations of PYC_*Re*_ enzyme (ATP + pyruvate + HCO_3_^−^ + H^+^  → ADP + phosphate + oxaloacetate) was introduced into the iML1515 model, and the metabolic response flux boundaries for the *tdcC*, *tdh*, *sstT*, *ilvA*, *poxB*, *pflB*, *pykF*, and *ldhA* genes were set to 0 for simulating gene knockout. FBA was used to simulate the allocation of intracellular carbon fluxes under conditions that maximized l-threonine biosynthesis (Fig. 6A) [36]. In silico simulation indicated an upregulation of glutamate dehydrogenase (encoded by the *gdhA* gene) when maximizing l-threonine production, suggesting the amino donor provided by l-glutamate was a crucial limiting factor for l-threonine synthesis. To ensure rational expression of GdhA, a reporter system for indirect quantification of *gdhA* gene expression level was developed by ligating the first 210 bp of the *gdhA* gene to eGFP (Fig. 6B)*.* Subsequently, a ribosome-binding site (RBS) library of *gdhA* gene was generated, resulting in RBS mutations that increased reporter gene expression by 1.2–3.5 folds compared to the original P_*trc*_-_RBS_ combination (Fig. 6C). Substituting the native promoter of the *gdhA* gene with different P_*trc*_-_RBS_ combinations, resulting an increase and then a decrease in l-threonine production with the GdhA expression level. In addition, it was observed that 1.2 g/L and 2.3 g/L of l-glutamate were accumulated in the RBS4 and RBS5 mutants, respectively, with no significant change in biomass (Fig. S4). THRM7 strain, overexpressing *gdhA* with the promoter P_*trc*_-_RBS3_, resulted in 44.12 g/L l-threonine. This indicated that it was essential to carefully regulate GdhA expression to avoid limiting l-threonine synthesis due to insufficient l-glutamate supply, while also preventing a decrease in l-threonine production due to excessive l-glutamate accumulation.

Subsequently, in silico five engineered targets were predicted that needed to be knocked out under conditions of maximized l-threonine production by combining the extended GSMN with the OptKnock software. These five genes (*ydbk*, *ompN*, *aroL*, *phoA*, *puuE*) were individually knockout in THRM7 strain to produce THRM8 (Δ*ydbk*), THRM9 (Δ*ompN*), THRM10 (Δ*aroL*), THRM11 (Δ*puuE*), THRM12 (Δ*phoA*). As shown in l-threonine production increased by 1.63% and 2.17% when the *ompN* and *phoA* genes were knockout, respectively. By further deleting the *ompN* gene in the THRM12 strain, the THRM13 strain was generated, achieving an l-threonine yield of 46.02 g/L. Finally, the fermentation performance of the THRM13 strain was validated through fed-batch fermentation in a 5 L bioreactor, achieving an accumulation of 163.2 g/L, 0.603 g/g glucose (Fig. 6E). This is the highest level of l-threonine production reported so far in *E. coli*. These findings advance our understanding and offer novel strategies for enhancing aspartic acid family derivative production.

## Conclusions

In this study, a highly sensitive and high fluorescence threshold biosensor was developed to assist the high-throughput platform to screen for superior mutants. Subsequently, cellular metabolic flow distribution was optimized and l-threonine production was further enhanced by multi-omics analysis guided reverse metabolic engineering and in silico simulations. Ultimately, engineered strain, THRM13, demonstrated performance by producing 163.2 g/L of l-threonine in a 5 L bioreactor, with a yield of 60.3%. This represents the highest reported l-threonine production without the use of exogenous inducers and antibiotics. In addition, this high-throughput screening strategy could still be used for subsequent iterative evolution of strains.

## Supplementary Information

Supplementary Material 1.

## Data Availability

Data is provided within the manuscript or supplementary information files.
