# Supplementary material for: Combining biosensor and metabolic network optimization strategies for enhanced l-threonine production in Escherichia coli
Source: Biotechnol Biofuels Bioprod. 2025 Mar 26;18:37. doi: 10.1186/s13068-025-02640-7 (PMC11938683; doi:10.1186/s13068-025-02640-7)
Supplement: Supplementary file 1 — Supplementary Material 1. [file 13068_2025_2640_MOESM1_ESM.docx]

**Figure S1.** **Negative and positive control experiments in biosensor development**


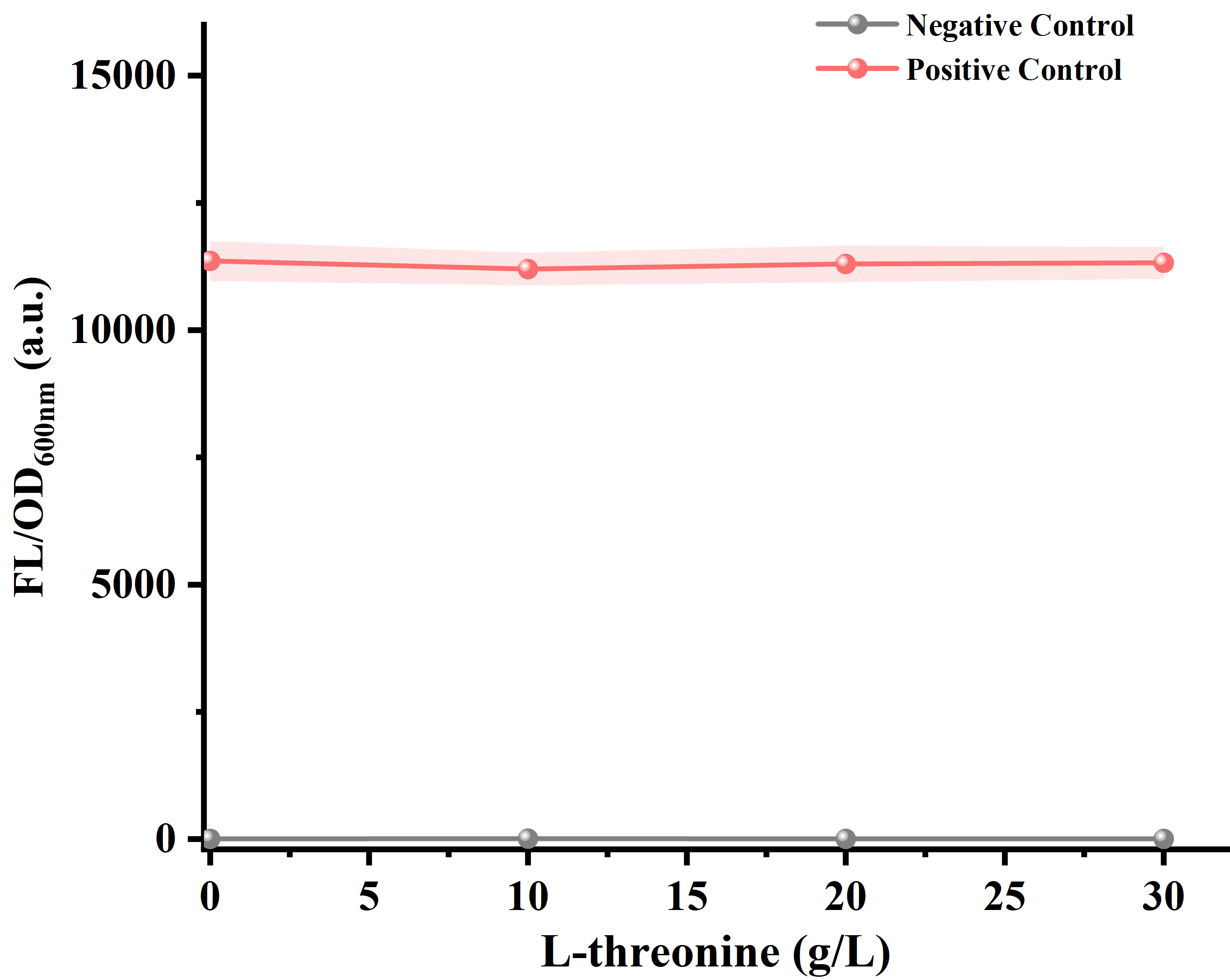


**Figure S2. Intracellular and extracellular L-threonine concentrations and biosensor response performance during fermentation. Changes in fluorescence intensity and intracellular/extracellular L-threonine concentrations in THR36-L19-pSensorThr strain during fermentation at 0, 2, 4, 6, 8, 10, and 12 hours (ND: Not Detected)**


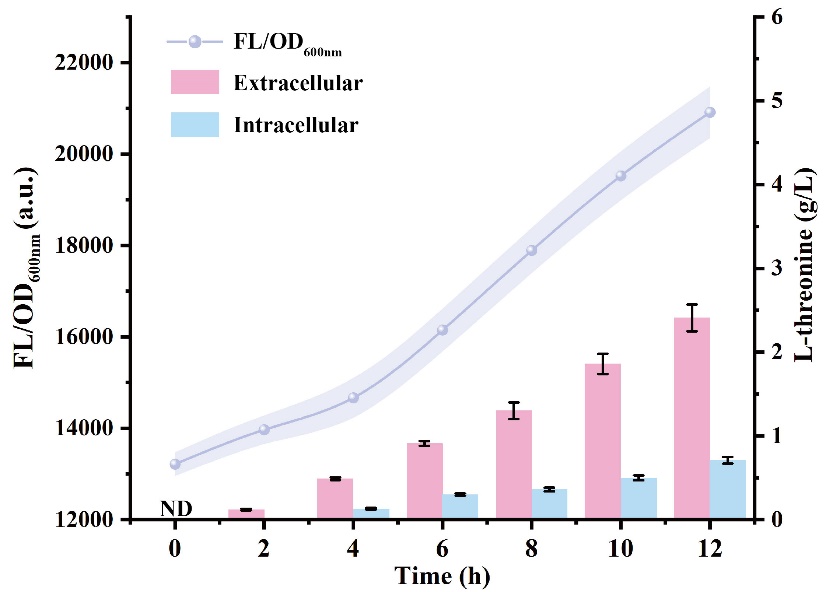


**Figure S3.** **The L-threonine production and cell growth of THRM3 and THRM4~6 strains in shake flask cultivation.**


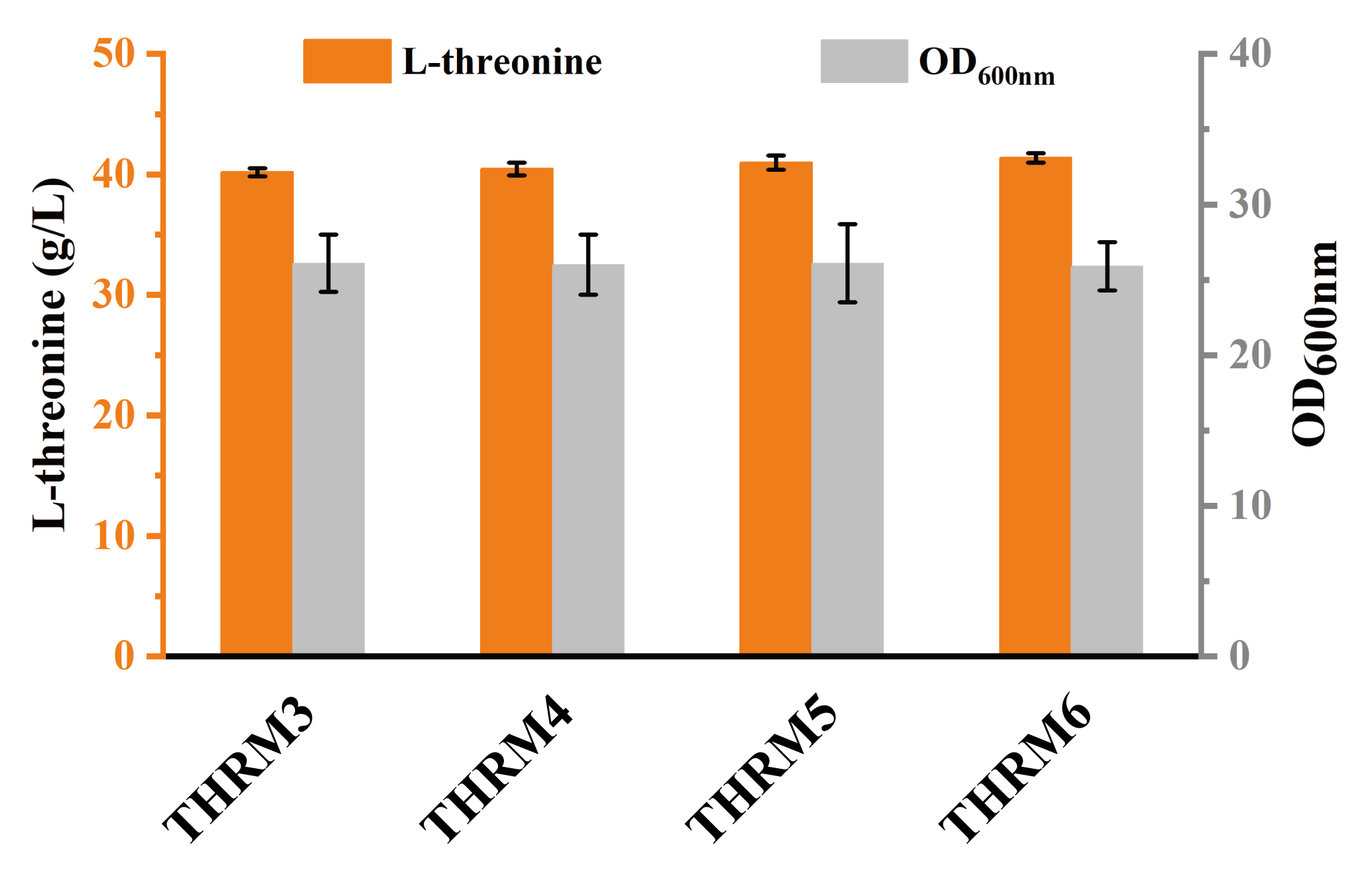


**Figure S4.** **Impact of optimizing *gdhA* expression on L-glutamate accumulation and strain growth.**


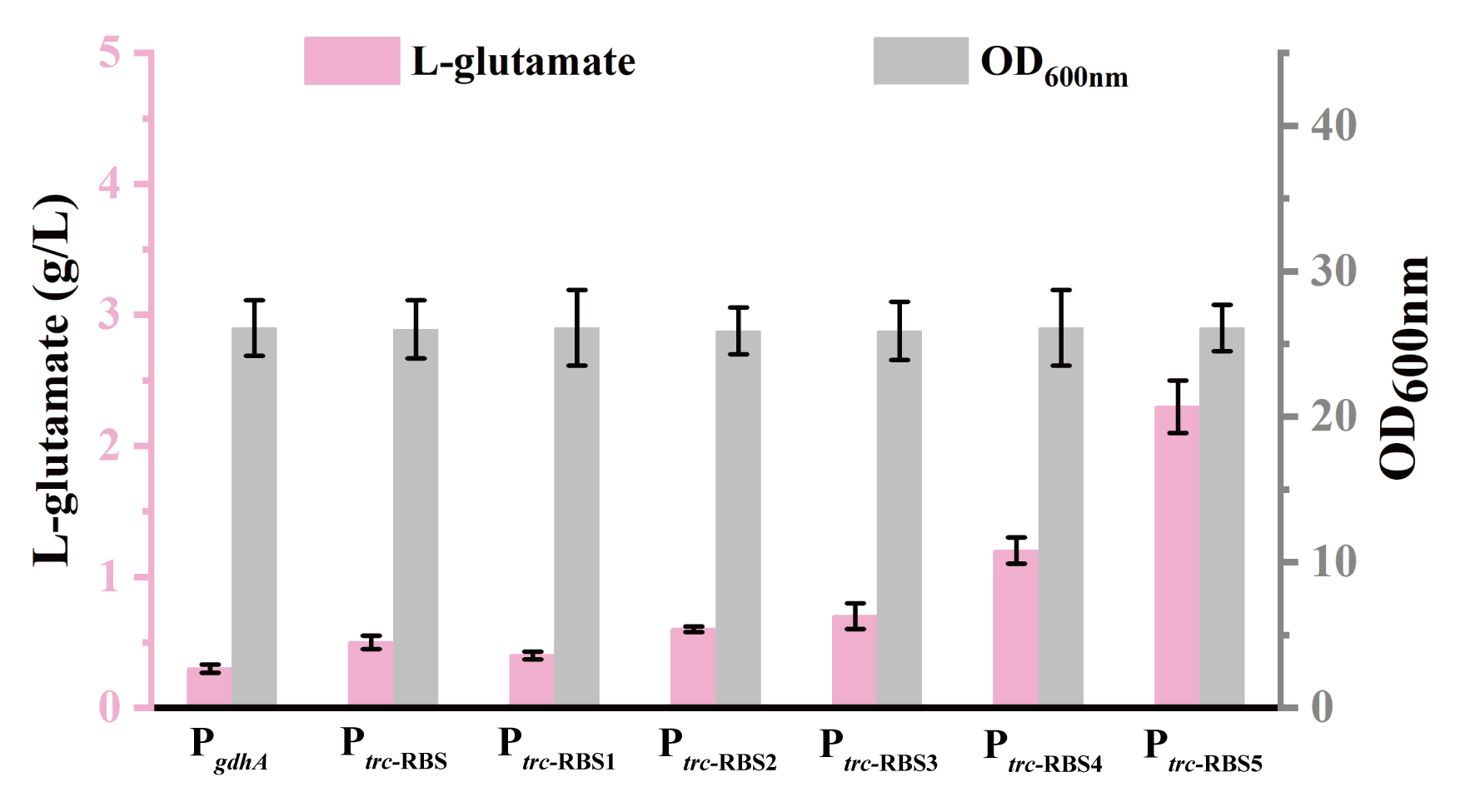


**Table S1. Comparison of L‐threonine production in engineered *Escherichia coli* strains.**

| **Strain** | **Titer**  **(g/L)** | | **Yield**  **(g/g)** | | **Productivity (g/L/h)** | | | **References** |
| --- | --- | --- | --- | --- | --- | --- | --- | --- |
| *E. coli* EC125 | | 105.3 | | 0.405 | | 2.194 | (Liu et al. 2013) | |
| *E. coli* MT201 | | 102 | | 0.375 | | 1.7 | (Lee et al. 2018) | |
| *E. coli* JLTHR | | 127.3 | | 0.581 | | 4.243 | (Su et al. 2018) | |
| ThrH27(pTZL2) | | 123.61±3.07 | | 0.46 | | 2.63±0.07 | (Liu et al. 2015) | |
| TWF083 | | 116.62 | | 0.486 | | 2.43 | (Zhao et al. 2020) | |
| *E. coli* TH-103Z | | 160.3 | | 0.55 | | 1.66 | (Wang et al. 2023) | |
| *E. coli* THRM13 | | 163.2 | | 0.603 | | 3.4 | This study | |

**Table S2. Strains and plasmids used in this study.**

| **Strain** | **Description** | **Sources** |
| --- | --- | --- |
| *E. coli* MG1655 | Wild-type strain | This lab |
| *E. coli* DH5α | Cloning host | This lab |
| THR01 | wild-type *E. coli* MG1655, Δ*lacI*, Δ*tdh* | This lab |
| THR36-L19 | L-threonine-producing strain | This lab |
| THR36-L19  (Tpx C61G) | THR36-L19 derivative with amino acid exchange C61G in the *tpx* gene | This work |
| THR36-L19 (SpoT R290H) | THR36-L19 derivative with amino acid exchange R290H in the *spoT* gene | This work |
| THR36-L19 (DeoB C350Y) | THR36-L19 derivative with amino acid exchange C350Y in the *deoB* gene | This work |
| THR36-L19 (TrmC A129P) | THR36-L19 derivative with amino acid exchange A129P in the *trmC* gene | This work |
| THR36-L19 (*cadA^A400T^*) | THR36-L19 derivative with nucleotide exchange A400T in the *cadA* gene | This work |
| THR36-L19 (*yeeI^T188A^*) | THR36-L19 derivative with nucleotide exchange T188A in the *yeeI* gene | This work |
| THRM1 | THR36-L19 derivative generated by MP6^ts^ mutagenesis | This work |
| THRM2 | THRM1 derivative with amino acid exchange G61C in the *tpx* gene | This work |
| THRM3 | THRM1 derivative with amino acid exchange H290R in the *spoT* gene | This work |
| THRM4 | THRM3, Δ*pykF* | This work |
| THRM5 | THRM4, Δ*poxB* | This work |
| THRM6 | THRM5, Δ*pflB* | This work |
| THRM6 (P*_gdhA_*::P*_trc_*_-RBS1_) | THRM6, replacing the *P_gdhA_* to *P_trc_*_-RBS1_ | This work |
| THRM6 (P*_gdhA_*::P*_trc_*_-RBS2_) | THRM6, replacing the *P_gdhA_* to *P_trc_*_-RBS2_ | This work |
| THRM6 (P*_gdhA_*::P*_trc_*_-RBS4_) | THRM6, replacing the *P_gdhA_* to *P_trc_*_-RBS4_ | This work |
| THRM6 (P*_gdhA_*::P*_trc_*_-RBS5_) | THRM6, replacing the *P_gdhA_* to *P_trc_*_-RBS5_ | This work |
| THRM7 | THRM6, *P_gdhA_*::*P_trc_*_-RBS3_ | This work |
| THRM8 | THRM7, Δ*ydbK* | This work |
| THRM9 | THRM7, Δ*ompN* | This work |
| THRM10 | THRM7, Δ*aroL* | This work |
| THRM11 | THRM7, Δ*puuE* | This work |
| THRM12 | THRM7, Δ*phoA* | This work |
| THRM13 | THRM12, Δ*ompN* | This work |
| **Plasmid** | **Description** | **Sources** |
| pREDCas9 | CRISPR-Cas9 system, carrying Cas9 protein and λ Red system, temperature sensitive | (Lin et al. 2015) |
| pGRB | CRISPR-Cas9 system, for expression of gRNA in *E. coli* | (Lin et al. 2015) |
| pTrc99A | Expression vector of *E. coli*, IPTG-inducible promoter *P_trc_*, Amp^R^ | MIAOLING BIOLOGY |
| pTrc99A-P*_cysK_ -egfp* | pTrc99A backbone, replacing the *P_trc_* promoter with *P_cysk_-egfp* | This work |
| pSensor | pTrc99A backbone, carrying the *P_cysK_ -egfp* gene and CysB protein | This work |
| pSensorThr | pTrc99A backbone, carrying the *P_cysK_ -egfp* gene and CysB T102A protein | This work |
| pTrc99A-*gdhA-egfp* | pTrc99A derivative carrying the *gdhA-egfp* gene | This work |
| pTrc99A-RBS1-*gdhA*-*egfp* | pTrc99A-*gdhA-egfp* derivative replacing the original RBS with RBS1 | This work |
| pTrc99A-RBS2-*gdhA-egfp* | pTrc99A-*gdhA-egfp* derivative replacing the original RBS with RBS2 | This work |
| pTrc99A-RBS3-*gdhA-egfp* | pTrc99A-*gdhA-egfp* derivative replacing the original RBS with RBS3 | This work |
| pTrc99A-RBS4-*gdhA-egfp* | pTrc99A-*gdhA-egfp* derivative replacing the original RBS with RBS4 | This work |
| pTrc99A-RBS5-*gdhA-egfp* | pTrc99A-*gdhA-egfp* derivative replacing the original RBS with RBS5 | This work |

**Table S3. promoter sequences used in this study (containing RBS sequences).**

| **Promoter** | **sequence** |
| --- | --- |
| P*_trc_* | TTGACAATTAATCATCCGGCTCGTATAATGTGTGGAATTGTGAGCGGATAACAATTTCACACAGGAAACAGACC |
| P*_trc-_*_RBS1_ | TTGACAATTAATCATCCGGCTCGTATAATGTGTGGAATTGTGAGCGGATAACAATTTCACTGAAGGAAGTGACC |
| P*_trc-_*_RBS2_ | TTGACAATTAATCATCCGGCTCGTATAATGTGTGGAATTGTGAGCGGATAACAATTTCACACCAGAGTCAGACC |
| P*_trc-_*_RBS3_ | TTGACAATTAATCATCCGGCTCGTATAATGTGTGGAATTGTGAGCGGATAACAATTTCACCAAGAGAGTAGACC |
| P*_trc-_*_RBS4_ | TTGACAATTAATCATCCGGCTCGTATAATGTGTGGAATTGTGAGCGGATAACAATTTCACGAAGTGCATAGACC |
| P*_trc-_*_RBS5_ | TTGACAATTAATCATCCGGCTCGTATAATGTGTGGAATTGTGAGCGGATAACAATTTCACCTAGAAGTCAGACC |
| P*_cysD_* | AGTGAGTCCTTAAATACCATGCAAATTTTTTTACCGCCATAGTATGAAACTGCCGCTGCGCTAAAACAATTTCAAATCTTCCTAAACGCCCGAAATCCGGTGCCTTAAGCACTTTTTGATATTAGCTTTGCCAAATCGTTATTCCGTTAAGGAACTACTCATTCTAATTGGTAATTTCATTCGTTCTCTTACGCTCCCTATAGTCGAAACATCTGATGGCAAGAAAATAGCGGTATTGCAAAGGAACGGTT |
| P*_cysP_* | TTCACCTCTGTTTCCTCCCTGCATTTGTGGGGAGGATTTCGTCTTGAACTAAGTTCACCAGGCTATTTTATTTGTCATTTTGGCCCCGGGCAGTGCTCGAAATCCTCACGTACTATGTGTACGCTCCGGTTTCTCCGCGCTGTTCGTGTCCAAACTGACTGCAACAATTACGCCTGTTGAACCAAGTTCTTATTCCCTTTTCAACTTCCAAATCACCAAACGGTATATAAAACCGTTACTCCTTTCACGTCCGTTATAAATATGATGGCTATTAGAAAGTCATTAAATTTATAAGGGTGCGCA |
| P*_cysK_* | CAATCTACCGGTTATTTTGTAAACCGTTTGTGTGAAACAGGGGTGGCTTATGCCGCCCCTTATTCCATCTTGCATGTCATTATTTCCCTTCTGTATATAGATATGCTAAATCCTTACTTCCGCATATTCTCTGAGCGGGTATGCTACCTGTTGTATCCCAATTTCATACAGTTAAGGACAGGCC |
| P*_cysJ_* | AATTTCTCTACAGGACGATAAAACCGCCGTAGAGTACCGGAAAGGCTGTTTTTTCGCATTATCTAAAACGACTTTGTTGCGCAAAATCGCTGATTTATCTTAATGATTGGCTAAATTCATTTGTTTTTCATTAGGTTGGTTAATCTATTTTGTTGTTAAAGACTATTGCTAAAACAGGTTAGTCGATTTGGTTATTAGTTATCGCTATCCCGTCTTTAATCCACACCGTTTGCCCCGTTAACCTTACCTTCTCTTCTGTTTTATGGGCGCTGACAGGGCGCAGAAACAGCTTTGCTTACTGGAACATAACGACGC |
| P*_sbp_* | TGATTTCGGAAAAAGGCAGATTCCTTTACCCTGAAACCGATGACAGAAGCAAAAATGCCTGATGCGCTTCGCTTATCAGGCCTACATGAATTCTGCAATTTATTGAATTTGCAAACTTTTGTAGGCCGGATAAGGCGTTCGCGCCGCATCCGGCATGGACAAAGCGCACTTTGTCAGCAATATGAGGCGGATTTCTTCCGCCTTTTTAATCCCTCAACATATACCCGCAAGTTATAGCCAATCTTTTTTTATTCTTTAATGTTTGGTTAACCTTCTGGCACGCTTTGCTCATCACAACACAACATAAGAGAGTCGGGCG |
| P*_cysH_* | CCATCAGCCCGGTCTTGTAGGCCTGATAAGAACGCGTGAGCGTCGCATCAGGCAAGGCAAACAGTGAGGAATCT |
| P*_yciW_* | TCGTTCTGTTGGTAAAGATGGGCGGCGTTCTGCCGCCCGTTATCTCTGTTATACCTTTCTGATATTTGTTATCGCCGATCCGTCTTTCTCCCCTTCCCGCCTTGCGTCAGGATAACGATTTCCTTTACGACCAAGGAGCGCCC |
| P*_fliY_* | ATAAGAATAAGATGTAGCGGAGTTGTTTTTGTGTTTACAAACAATGGCTCTACACTGCAAACAGACATAACAACATTCGGGGTGAAT |
| P*_ygeW_* | TATCAATATGAGAATTACGGCGGTGAGTTTATCAAACTGAAGAGAGATAGCCTGCCCCTTTATCTTATTTCTGATACTTAGCAGCAAATAAATAACGCGATAAAAAAAGCCAAACGTTTTCGTATTTTACAAACAACCAGAAGCTGGCATCAATTTGTGATCAACCCCACACATTATCCGTCAAATTAGTCTTTTGCAGCCGCGCGGATAATTCTGGCACACTTATTGTTAGTCCCAGGTATAGCTGTGAAAACACCAATCACTTTGGCAAGTCACAGTGAAATAAACCACTTTGCCTGTCATTCCACTACCGGGACTTT |
| P*_srlA_* | GAGGGGTCCATTTAACAGATTCAACCAGGGGCAAGTATGGTAAAGCATCACGCCCCGCACAAGGAAGCGGTAGTCACTGCCCGATACGGACTTTACATAACTCAACTCATTCCCCTCGCTATCCTTTTATTCAAACTTTCAAATTAAAATATTTATCTTTCATTTTGCGATCAAAATAACACTTTTAAATCTTTCAATCTGATTAGATTAGGTTGCCGTTTGGTAATAAAACAATAAATCCTGAAGGAGAGAACA |
| P*_yfiD_* | TTGGTAAAATTTCACACCCGCATAAGTTGATGTAAAACAATAAAATCCATTTGCGACCATACTTGATGTGTGGTTTTTATTGATTTAAATCAAAGATTCAAGGGTGTTTGAGGAGTATATATACACTCAAGCAACAATGGTTTTACCAATTGGCCGCGACAGGCTGAACAAATCAAATAATTTTGCCGGGGAGGCATCAC |
| P*_pdhR_* | TCAACCCCTCTCAATATGTAGAATGAATTTAAATTCGTTTTAATTGAATTAAAAATCACAAAATTGGTAAGTGAATCGGTTCAATTCGGATTTTTATAGTTTAATAATCGTTAAAAAACTCCTTTCCTACGTAAAGTCTACATTTGTGCATAGTTACAACTTTGAAACGTTATATATGTCAAGTTGTTAAAATGTGCACAGTTTCATGATTTCAATCAAAACCTGTATGGACATAAGGTGAATACTTTGTTACTTTAGCGTCACAGACATGAAATTGGTAAGACCAATTGACTTCGGCAAGTGGCTTAAGACAGGAACTC |
| P*_yeeE_* | TCATCTTTTCTAATAATAAACCGGAGTCGAATGAACATTTGGCTCCGGTTTTTTTATTCTTTTGTATCGAACTCCTCATTAATAACCATTTGTTCTAATTTAGAGAATTTGGTTATTGGTCTGCATTCCACCAGTCATGAATAATTTCCTCATTAACATTCATCTGGTTAATTAATTT |
| P*_nlpA_* | GGATTCAACCTTTTACAAATGTGTATCGAATGAAGCATAAGTGTAGTTTGCTTTCATCGCTCTCATCATGCTGTACGAAACGCGCTTGCGTGGTCAGTAAGAAGTGCCAGACTTTATATTCCACTTTTATTCCTTTTTATTCTTATCGATAGCGTTTCGTTTTTTAAACCGCAGCGACCTTACCGCTATAGTCAGGTAATCATTAATAAAAGGATAAAAAA |
| P*_xdhA_* | TGCGTAATAATCCCAATTTATCACATTGATTTTATCGCCACTCATATTGATACGTATCACTGCCCCTATTCAGAGTCCTGTTTCGTATCATATCTGTGTTTTTTTAACGTCATCACACTTCCTACCTCCCCTAACGCTTATCGTCGTTTCTGGCGTAAATCTTGCCTGCTTAGACTAAATCTTTGCCATAAGAACCAAATGTTCAAATCGAGGGGATTTCTATGGAAGCGCGGGAAGCAACCGCTACGGGTGAATCATGC |
| P*_proV_* | TTGACAAAAAATATCAACTTTCTCGATTTGCTCTCAGCCCTTATATCACGGGAAATTCCGGCGATTTGCTCGCATCAATATTCATGCCACATTTGCCATCAGGGGTTGCCTCAGATTCTCAGTATGTTAGGGTAGAAAAAAGTGACTATTTCCATTGGGTAATATATCGACATAGACAAATAAAGGAATCTTTCTATTGC |
| P*_ndh_* | TGTTTTTTGATCTCACCCGGTAAAGTCGCCTATCTTTTCAGCAACAAAACTTGATTAACATCAATTTTGGTATGACCAATGCACCATTCATGTTATTCTCAATAGCGAAGAACATTTTCATTGCTGTAACCTGTTGTTAATTAAGAGCTATGTTAATAACCATTAATTAACAATTGGTTAATAAATTTAAGGGGGTCACG |
| P*_ydeN_* | ATTAAATATCAATAAATTAGTTGTTTATCGGCGAGAAATTACTTAATAGAACAGAAAGTAATGTCAACGCTTTATGGACTGTTTTTTCCCTTTTTTTAGCTAAATCTGCTATCTCTTTATGTGACTAACTTCACTTACATCCACTTATTTCTCTTCGTAAAATTACTTTGGAATTAAGTACAATAAGAAGAGGAACATTT |
| P*_yghZ_* | TCCAGCAACTCTTGTGGGAAATCTTTGGCGGTTAAACGCGGCATCGTTGCACTCCTCAGTTGGTGTTTTTTTAAGCAAAGCATAAGCACGTATTTTTGCCCAGTTTTTCGTCACTCTGTGAGCCAGACTACGGGATACGCGCTGGCGAATCGCTAAACTAGAAACATTGTTTCGAAATTGAACGGTGGAAAGGAGAGGTC |
| P*_ygfK_* | TTTCAGGAGTGATTTTAATTCTCATTTAGCACCTGTGAGTTCATTCACAAAAAGCCCTTAAGTTTCGAGCCGCCTCACATTTTTTTATATTTCCCGCCAAACCTGGCAAGAGTGGTGCGATTGTTGCTCTATCCCCCTAAACCACCGGATTTCTCAACACCGGTCACTCAATGATATCTGTATAAGCTAAGGAGAGGGTT |
| P*_ynfM_* | ATTTAAAACATCTTATTTGAGATTATTAATATATTAGACAGAACAATTCGATTTTCCTACCCTATGTATAAGCCTGATCTACAGGCATATTTAGCAAGGATTTCAA |

The marked red font is the mutated region of RBS

**Table S4.** **Genetic information of mutants in the THRM1 strain**

|  |
| --- |

| **Gene** | | **sizes** | **DNA Mutation** | | **Protein Mutation** |
| --- | --- | --- | --- | --- | --- |
| *tpx* | 507 | | | T181G | C61G (TGC**→**GGC) |
| *deoB* | 1224 | | | G1049A | C350Y (TGC**→**TAC) |
| *trmC* | 2007 | | | G385C | A129P (GCG**→**CCG) |
| *spoT* | 2109 | | | G869A | R290H (CGC**→**CAC) |
| *cadA* | 2148 | | | A400T | K134Stop (AAA**→**TAA) |
| *yeeI* | 798 | | | T188A | L63Stop (TTA→TAA) |

**References**

Lee, H.W., et al., 2018. Enhancement of L-hreonine production by controlling sequential carbon-nitrogen ratios during fermentation. J. Microbiol. Biotechnol. 28(2), 293-297.

Lin, et al., 2015. Metabolic engineering of *Escherichia coli* using CRISPR-Cas9 meditated genome editing. Metab. Eng. 31, 13-21.

Liu, S., et al., 2013. Development of a two-stage feeding strategy based on the kind and level of feeding nutrients for improving fed-batch production of L-threonine by *Escherichia coli*. Appl. Microbiol. Biotechnol. 97(2), 573-583.

Liu, Y., et al., 2015. Developing a high-throughput screening method for threonine overproduction based on an artificial promoter. Microb. Cell. Fact. 14, 121.

Su, Y., et al., 2018. Effects of betaine supplementation on L-threonine fed-batch fermentation by *Escherichia coli*. Bioprocess. Biosyst. Eng. 41(10), 1509-1518.

Wang, S., et al., 2023. Creating polyploid *Escherichia coli* and its application in efficient L-threonine production. Adv. Sci. (Weinh) 10(31), e2302417.

Zhao, L., et al., 2020. Expression regulation of multiple key genes to improve L-threonine in *Escherichia coli*. Microb. Cell. Fact. 19(1), 46.
